# *Populus tomentiglandulosa* Extract Is Rich in Polyphenols and Protects Neurons, Astrocytes, and the Blood-Brain Barrier in Gerbil Striatum Following Ischemia-Reperfusion Injury

**DOI:** 10.3390/molecules26185430

**Published:** 2021-09-07

**Authors:** Tae-Kyeong Lee, Jae-Chul Lee, Jong-Dai Kim, Dae-Won Kim, Ji-Hyeon Ahn, Joon-Ha Park, Hyung-Il Kim, Jun-Hwi Cho, Soo-Young Choi, Moo-Ho Won, II-Jun Kang

**Affiliations:** 1 Department of Biomedical Science, Research Institute of Bioscience and Biotechnology, Hallym University, Chuncheon 24252, Gangwon, Korea; tk_lee@hallym.ac.kr (T.-K.L.); sychoi@hallym.ac.kr (S.-Y.C.); 2 Department of Neurobiology, School of Medicine, Kangwon National University, Chuncheon 24341, Gangwon, Korea; anajclee@kangwon.ac.kr (J.-C.L.); jh-ahn@ysu.ac.kr (J.-H.A.); 3 Division of Food Biotechnology, School of Biotechnology, Kangwon National University, Chuncheon 24341, Gangwon, Korea; jongdai@kangwon.ac.kr; 4 Department of Biochemistry and Molecular Biology, Research Institute of Oral Sciences, College of Dentistry, Gangnung–Wonju National University, Gangneung 25457, Gangwon, Korea; dwkim@hallym.ac.kr; 5 Department of Physical Therapy, College of Health Science, Youngsan University, Yangsan 50510, Gyeongsangnam, Korea; 6 Department of Anatomy, College of Korean Medicine, Dongguk University, Gyeongju 38066, North Gyeongsang, Korea; jh-park@dongguk.ac.kr; 7 Department of Emergency Medicine, Dankook University Hospital, College of Medicine, Dankook University, Cheonan 31116, Chungnam, Korea; hilovesjj@naver.com; 8 Department of Emergency Medicine, Kangwon National University Hospital, School of Medicine, Kangwon National University, Chuncheon 24289, Gangwon, Korea; cjhemd@kangwon.ac.kr; 9 Department of Food Science and Nutrition, Hallym University, Chuncheon 24252, Gangwon, Korea

**Keywords:** astrocyte endfeet, BBB leakage, gliosis, immunoglobulin G, ischemia-reperfusion injury, neuronal damage/death

## Abstract

Transient ischemia in brains causes neuronal damage, gliosis, and blood–brain barrier (BBB) breakdown, which is related to ischemia-induced brain dysfunction. *Populus* species have various pharmacological properties including antioxidant and anti-inflammatory activities. In this study, we found that phenolic compounds were rich in *Populus tomentiglandulosa* extract and examined the effects of *Populus tomentiglandulosa* extract on neuronal damage/death, astrogliosis, and BBB breakdown in the striatum, which is related to motor behavior, following 15-min transient ischemia in the forebrain in gerbils. The gerbils were pre-treated with 50, 100, and 200 mg/kg of the extract. The latter showed significant effects against ischemia-reperfusion injury. Ischemia-induced hyperactivity using spontaneous motor activity test was significantly attenuated by the treatment. Striatal cells (neurons) were dead at five days after the ischemia; however, pre-treatment with the extract protected the striatal cells from ischemia/reperfusion injury. Ischemia-induced reactive astrogliosis was significantly alleviated, in particular, astrocyte end feet, which are a component of BBB, were significantly preserved. Immunoglobulin G, which is not found in intact brain parenchyma, was apparently shown (an indicator of extravasation) in striatal parenchyma at five days after the ischemia, but IgG leakage was dramatically attenuated in the parenchyma by the pre-treatment. Based on these findings, we suggest that *Populus tomentiglandulosa* extract rich in phenolic compounds can be employed as a pharmaceutical composition to develop a preventive material against brain ischemic injury.

## 1. Introduction

It has been demonstrated that the genus *Populus* (poplar) contains phenolic compounds and flavonoids which display various pharmacological activities [1]. *Populus davidiana* and *Populus nigra* possess pharmacological actions including anti-inflammatory, antioxidant, and hepatoprotective properties [2,3]. In addition, *Populus balsamifera* has pharmacological potential to treat diabetes and obesity [4]. Among *Populus* species, however, few studies about scientific validation of pharmacological effects of *Populus tomentiglandulosa* (Korean poplar) have been reported; only that *P. tomentiglandulosa* extract has neuroprotective effects in gerbil hippocampus against ischemia-reperfusion injury induced by 5-min transient ischemia in the forebrain [5].

It is accepted that brief transient ischemia in the brain causes selective neuronal damage/death (loss) in vulnerable regions due to ischemia [6,7]. Among the vulnerable regions, the hippocampus (proper), which consists of cornu ammonis 1 to 3 (CA1–3) subregions, has a CA1 region that is most vulnerable to transient ischemia [8,9]. Besides, the striatum, which is a part of the basal ganglia, is known as a vulnerable region to transient ischemia.

A gerbil model of transient forebrain ischemia (tFI) has been used to investigate the pathophysiology of injury induced by tFI and to develop new materials for the treatment of ischemic stroke [10,11,12]. Gerbils show the significant possibility of reproduction of tFI by occlusion of both common carotid arteries because they lack the posterior communicating arteries that connect the internal carotid and vertebral arteries in the Willis’s circle [13]. We reported that, in gerbils, the pattern of neuronal death in ischemic striatum and hippocampus was very different when we gave various durations (five to 20 min) of tFI [14,15]. The striatum is the largest nucleus of the basal ganglia, which receives glutamatergic inputs from the cerebral cortex and inputs containing dopamine from the substantia nigra [16,17]. It has been studied that the dorsolateral striatum is susceptible to transient brain ischemia in rats [18] and gerbils [19]. In addition, some researchers have demonstrated that, among neurons located in the striatum, spiny neurons of medium size are the most vulnerable to transient ischemia [17,20].

BBB, as an essential structure for brain homeostasis, is a highly selective barrier to restrict the transportation of substances between blood and central nerve system (CNS), which helps to sustain a healthy microenvironment in the brain [21]. BBB tightly regulates the movement of ions, molecules, and cells between blood and parenchyma, and, thus, is critical for neuronal function and protection. It has been reported that BBB breakdown following ischemic insults is one of the pathophysiological hallmarks of ischemic injury in the brain [22]. The evaluation of BBB permeability after ischemic insults is necessary for the development of effective strategies to prevent secondary brain injury or protect BBB function following the ischemic insults [23]. Until now, several methods to assess changes in BBB permeability following brain ischemia have been introduced [24]. Among them, the accumulation of immunoglobulin G (IgG) after brain ischemia is one of the widely used markers to determine BBB disruption [25,26,27,28]. In particular, astrocytes, in the CNS, form a BBB by supplying a link between blood vessels and neuronal circuitry, and, through such linkage, they provide nutrients to CNS tissue [29]. In addition, water balance and extracellular ion in the BBB are controlled by water channels and ions, which are expressed in astrocyte endfeet (AEF) [30].

However, neuronal damage, AEF damage, and BBB leakage in the striatum after severe transient forebrain ischemia (tFI) has not been fully reported. In addition, the effects of *Populus tomentiglandulosa* on neuronal damage, AEF damage, and BBB leakage following the ischemia have not been examined. Therefore, we analyzed phenolic compounds from *Populus tomentiglandulosa* extract and examined the effects of the extract on spontaneous motor activity, neuronal damage, AEF damage, and BBB leakage in the striatum following 15-min tFI in gerbils.

## 2. Materials and Methods

### 2.1. Preparation of Populus Tomentiglandulosa Extract (PTE)

As described previously [31], PTE was prepared as follows. In brief, the stem and root bark of *Populus tomentiglandulosa* inhabiting Gangwon-do (Republic of Korea) was harvested. It was washed with pure water, fully dried at 60 °C, and crushed into a fine powder using IKA M20 grinder (IKA, Staufen, Germany). Next, the powder was refluxed with 70% ethyl alcohol, which was ten times the volume of the powder at 70 °C for 24 h. The procedure of extraction was repeated three times. PTE was filtered using filter paper (Whatman No. 1 filter paper) (Whatman Ltd., Maidstone, Kent, UK) and concentrated in N-12 vacuum evaporator (Eyela Singapore Pte. Ltd., Singapore). Finally, using lyophilizer (FD8512) (ilShin BioBase Co. Ltd., Seoul, Republic of Korea), PTE was freeze-dried at −55 °C and stored at −20 °C. The yield of extraction was 14.7%.

### 2.2. Qualitative Analysis of PTE

To qualitatively analyze PTE, high-performance liquid chromatography (HPLC) was conducted in accordance with previously described methods [32,33]. In short, 10 mg of both test sample (PTE) and standard sample (phenolic compounds: caffeic acid, catechin, chlorogenic acid, ferulic acid, gallic acid, and *p*-coumaric acid; Sigma-Aldrich Co., St. Louis, MO, USA) was dissolved in 50% ethyl alcohol. Ten μL of each test and standard sample was chromatographed with 1.0 mL/min of flow rate for 90 min using Waters 2690 Separation Module HPLC System (Waters Co., Milford, MA, USA) and Sunfire™ C_18_ column (inner diameter, 4.6 mm; length, 250 mm) (Waters Co., Milford, MA, USA) filled with octadecylsilyl silica gel (diameter, 5 μm). A (acetonitrile) and B (phosphoric acid, H_3_PO_4_) solution were used as mobile phases with time-dependent A:B ratio as follows: 0–23 min (A, 8; B, 92), 23–40 min (A, 15%; B, 85%), 40–45 min (A, 30%; B, 70%), 45–62 min (A, 45%; B, 55%), 62–75 min (A, 45%; B, 55%), 75–82 min (A, 8%; B, 92%), and 82–90 min (A, 8%; B, 92%). The ingredients of PTE were determined at 278 nm of wavelength using Waters 996 Photodiode Array Detector (Waters Co., Milford, MA, USA).

### 2.3. Protocol, Experimental Animals and Groups

The protocol of the experiment including animal care and handling was approved (approval no. KW-2000113-1) on 13 January 2020 by Institutional Animal Care and Use Committee founded in Kangwon National University (South Korea). Gerbils were used in this study. They were cared under constant temperature (approximate 23 °C) and humidity (approximate 55%) with a 12-h light/dark cycle. The handling and caring of the gerbils conformed to the guidelines in the “Current international laws and policies” included in the “NIH Guide for the Care and Use of Laboratory Animals” from *The National Academies Press* (8th Ed., 2011).

**Figure 1:**
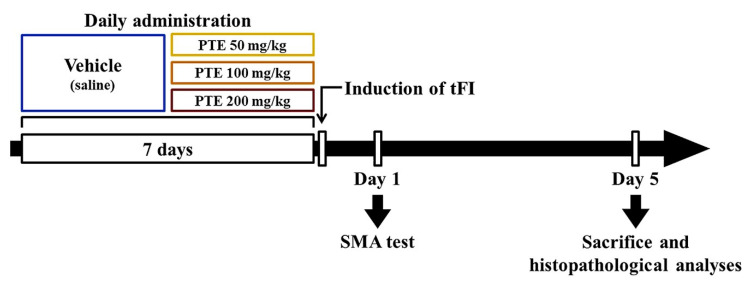
Experimental procedure. PTE is dissolved in saline, and orally administered once a day for 7 days before ischemic surgery. Experimental gerbils are sacrificed five days after 15-min transient forebrain ischemia (tFI) operation.

Male Mongolian gerbils at six months of age (body weight, 64–76 g) were received from the Experimental Animal Center founded in Kangwon National University (Chuncheon, Korea). The total number of the gerbils used in this study was 126, and they were assigned to four groups: 1) vehicle/sham group (subtotal *n* = 14), which was treated with saline (0.9% *w/v* NaCl) as vehicle and given sham tFI; 2) vehicle/tFI group (subtotal *n* = 14), which was treated with vehicle and given 15-min tFI; 3) PTE/sham group (subtotal *n* = 42), which was treated with 50, 100 and 200 mg/kg of PTE, respectively, and given sham tFI; 4) PTE/tFI group (subtotal *n* = 42), which was treated with 50, 100, and 200 mg/kg of PTE, respectively, and given 15-min tFI. PTE was dissolved in vehicle (saline), and vehicle or PTE was orally treated once a day for seven days before tFI operation (Figure 1). In this experiment, PTE was treated for one week, because various extracts obtained from plants are orally treated in traditional medicine, although no data on the absorption and metabolism of PTE have been reported (Figure 1).

### 2.4. Induction of tFI

As previously described in our published papers [34,35,36], the surgery for tFI was performed as follows. Briefly, the gerbils were given breathing anesthesia with 2.5% isoflurane mixed in 33% oxygen and 67% nitrous oxide. Under the anesthesia, both (right and left) common carotid arteries located in the neck were occluded for 15 min. After the occlusion, reperfusion (blood recirculation) was carried out under direct observation of retinal arteries using ophthalmoscope. Simultaneously, body temperature was controlled at normothermia (37 ± 0.5 °C) before tFI, during tFI, and after tFI. In this experiment, sham tFI was carried out, like the tFI surgery without the occlusion of both arteries.

### 2.5. Spontaneous Motor Activity (SMA) Test

In order to evaluate change in locomotor activity, the SMA test was performed during one hour at one day, as shown in Figure 1, after TFI according to a published procedure [14]. In short, the gerbils of each group were divided individually in a Plexiglas cage (25 cm × 20 cm × 12 cm), in which a soundproof chamber was located. Locomotor activity of each group was recorded using Photobeam Activity System-Home Cage (San Diego Instruments, San Diego, CA, USA). Each gerbil was observed continuously using a 4 × 8 photobeam, and the scores were checked from live observations. In addition, the video sequence of SMA test was used for subsequent re-analysis. In this test, times when each gerbil reared and times spent in grooming behavior were checked.

### 2.6. Preparation of Brain Sections for Histological Observation

Brain sections containing the hippocampus were prepared for histochemical and immunohistochemical changes at five days (at this time, neuronal death was shown in the striatum) after tFI in gerbils. In short, as described previously [37], the gerbils were deeply anesthetized with sodium pentobarbital (200 mg/kg, i.p.). Under the anesthesia, they were perfused transcardially with 4% paraformaldehyde solution, and their brains were removed. Immediately, the removed brains were more fixed in the same fixative for four hours. Thereafter, the brains were trimmed and cryoprotected by infiltration with 30% sucrose solution. For cryosection, the trimmed brains were frozen and serially sectioned into coronal sections (30-μm thickness) in cryostat of Leica (Wetzler, Germany).

### 2.7. Cresyl Violet (CV) Histochemistry

Histochemical staining with CV was performed in the hippocampus to examine distribution pattern of cells in all hippocampal subregions according to a published method [37]. Briefly, CV acetate (Sigma-Aldrich, St. Louis, MO, USA) was dissolved at 1.0% (*w/v*) in distilled water, and glacial acetic acid was added to this solution. The brain sections were stained with CV solution, and subsequently dehydrated by dipping in serial ethanol baths. Finally, the stained sections were mounted with Canada balsam (Kanto, Tokyo, Japan).

To examine the neuroprotective effect of PTE damage in ischemic striatum, five sections/gerbil in each group were taken and observed using microscope (BX53).

### 2.8. Histofluorescence with Fluoro-Jade B (F-JB)

Histofluorescence with F-JB was performed to investigate damage/death (loss) of cells in the hippocampus following tFI using published methods [38,39] with slight modification. In brief, the brain sections were incubated in potassium permanganate (KMnO_4_) solution (0.06%) (Sigma-Aldrich Co, St. Louis, MO, USA) for 10 min on a rotating stage. Immediately, the sections were rinsed in distilled water for 2 min and incubated in F-JB solution (0.0004%) (Histochem, Jefferson, AR, USA) for 20 min. After washing with distilled water, the sections were placed on a slide warmer for the reaction of F-J B and fully dried. Finally, the slides containing the sections were cleared by immersion in xylene (Junsei Chemical Co Ltd., Tokyo, Japan) and coverslipped with dibutyl phthalate polystyrene xylene (DPX) (Sigma-Aldrich Co, St. Louis, MO, USA).

For the count of F-JB positive cells (neurons), five sections per gerbil were analyzed, according to the method by Anderson et al. (2005), with some modification. In short, the sections containing F-JB positive cells were observed using an epifluorescence microscope (BX53) of Olympus (Tokyo, Japan) equipped with a 450–490-nm blue excitation light, and the F-JB positive cells were captured using image capture software (cellSens Standard) (Olympus, Tokyo, Japan). The captured F-J B positive cells were counted in 250 μm^2^ at the same areas in the striatum, and the mean number was calculated using NIH Image 1.59 software (NIH, Bethesda, MD, USA).

### 2.9. Immunohistochemistry (IHC)

In this experiment, IHC was performed to examine changes in astrocytes and BBB leakage according to a published method [40]. Briefly, we used rabbit anti-glial fibrillary acidic protein (GFAP) (diluted 1:1000; Merck Millipore, Temecula, CA, USA) for detection for astrogliosis, and rabbit anti-gerbil IgG (diluted 1:1000, Bioss antibodies, Atlanta, GA, USA) for detection for BBB leakage, as primary antibodies. The brain sections were incubated in each primary antibody solution at 4 °C for 10 h. Thereafter, these sections were reacted in the solution of biotinylated goat anti-rabbit IgG and streptavidin peroxidase complex (diluted 1:250, Vector, Burlingame, CA, USA). Finally, to change the end product into brown, the sections were reacted in solution of 3,3′-diaminobenzidine tetrahydrochloride (DAB) (Sigma-Aldrich Co, St Louis, MO, USA).

For a negative control in this experiment, the same tissues were incubated in pre-immune serum except for each primary antibody. In the sections, no structures with immunoreaction were found (data not shown).

To evaluate the changes in GFAP and IgG immunoreactivities, the relative optical density (ROD) of GFAP and IgG immunoreactive structures was applied. Five sections/gerbil were taken and observed using microscope (BX53) with a digital camera (DP72). In brief, as previously described [41], the images of GFAP and IgG immunoreactive structures were captured using software of cellSens Standard (Olympus, Tokyo, Japan). The captured images were converted into 8-bit grayscale images with a range of 0–255 (from black to white). Each image was assessed for grayscale intensity, and the immunoreactive intensity of the average staining was calculated using Image J software (version 1.46) (National Institutes of Health, Bethesda, Maryland, MD, USA). The immunoreactive intensity, as ROD, was relatively presented as a percentage (100% in the vehicle/sham group).

### 2.10. Double Immunofluorescence

In this study, double immunohistofluorescence was performed to distinguish AEF from endothelial cells in BBB using a method with some modification, as described previously [34]. In brief, as primary antibodies, mouse anti-GFAP (a marker for astrocytes) (diluted 1:1000, Chemicon, Temecula, CA, USA) and rabbit anti-glucose transporter 1 (GLUT-1, a marker for endothelial cells) (diluted 1:100, Chemicon) were reacted. After washing, these sections were reacted in the mixture of donkey anti-mouse IgG conjugated with Alexa Fluor^®^ 488 (diluted 1:500) (Invitrogen, Waltham, MA, USA) and goat anti-rabbit conjugated with IgG Alexa Fluor^®^ 546 (diluted 1:500) (Invitrogen, Waltham, MA, USA). Thereafter, they were rinsed, dehydrated in dry oven of WiseVen^®^ WOC High Clean Air Oven (Daihan Scientific Co Ltd., Gangwon, South Korea), and coverslipped with Canada balsam (Kanto Chemical Co., Inc., Tokyo, Japan).

The double immunoreaction (GFAP/GLUT-1) was observed using confocal MS (LSM510 META NLO) from Carl Zeiss (Oberkochen, Germany) located in the Korea Basic Science Institute Chuncheon Center (Chuncheon, Kangwon, South Korea).

### 2.11. Statistical Analysis

Data shown in this research represented the mean ± standard error of the mean (SEM) among experimental groups. The data were statistically analyzed using SPSS 18.0 (SPSS) (Chicago, IL, USA). The analysis of variance (ANOVA) with a post-hoc Bonferroni’s multiple comparison test was performed to determine differences among the groups. *p* < 0.05 was used for statistical significance.

## 3. Results

### 3.1. PTE Contained Phenolic Compounds

**Figure 2:**
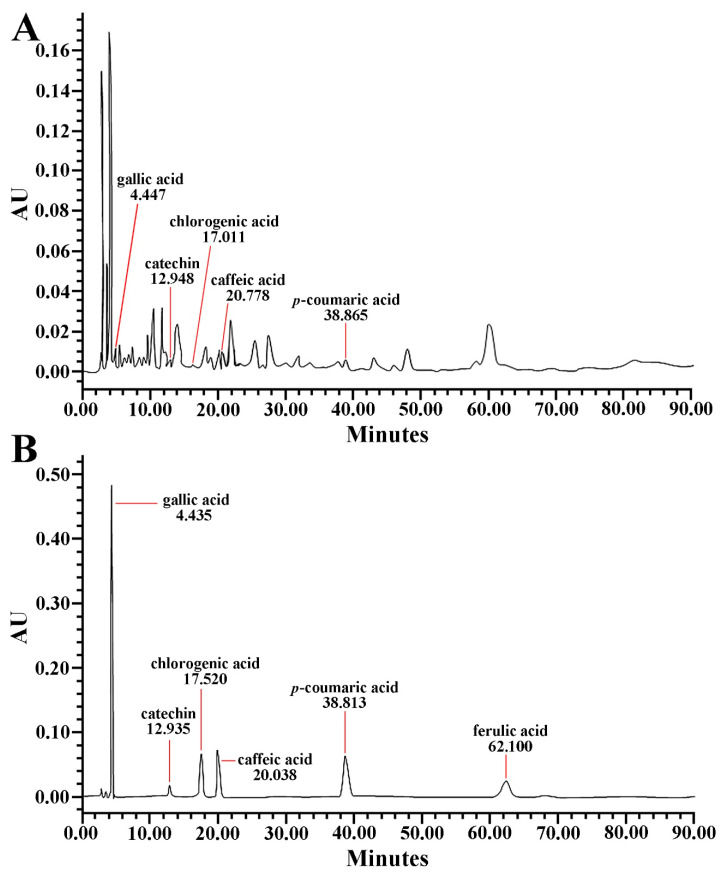
Representative HPLC chromatograms of standard (**A**) and PTE (**B**). The retention times of the standard samples (phenolic compounds) are 4.435 min (gallic acid), 12.935 min (catechin), 17.520 min (chlorogenic acid), 20.038 min (caffeic acid), 38.813 min (*p*-coumaric acid), and 60.100 min (ferulic acid). The retention times of the test sample (PTE) are 4.447 min (gallic acid), 12.948 min (catechin), 17.011 min (chlorogenic acid), 20.778 min (caffeic acid), and 38.865 min (*p*-coumaric acid).

As shown in the chromatograms for the test and standard samples, each phenolic compound (gallic acid, catechin, chlorogenic acid, caffeic acid, *p*-coumaric acid) contained in PTE was detected at 4.447, 12.948, 17.011, 20.778, and 38.865 min of latency time, respectively (Figure 2A,B). In addition, as tabulated in Table 1, each phenolic compound contained in PTE was determined.

### 3.2. PTE 200 mg/kg Was Significantly Effective on tFI-induced Locomotor Activity

**Figure 3:**
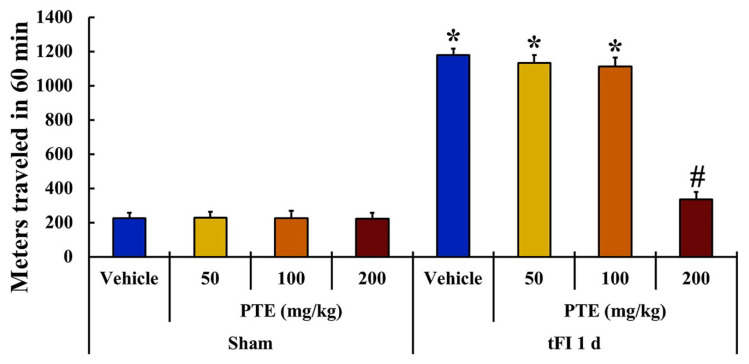
Changes in locomotor activity of the vehicle/sham, PTE (50, 100, and 200 mg/kg)/sham, vehicle/tFI, and PTE (50, 100, and 200 mg/kg)/tFI groups at 1 day after tFI. Among the PTE (50, 100, and 200 mg/kg)/tFI groups, 200 mg/kg of PTE is effective on locomotor activity after tFI (*n* = 7 per group; * p < 0.05, significantly different from vehicle/sham group, *^#^ p* < 0.05, significantly different from vehicle/tFI group). The *bars* indicate the means ± SEM.

Locomotor activity in all groups was evaluated by using a spontaneous motor activity test to compare the change in motor behavior at day 1 after tFI (Figure 3). Locomotor activity was similarly observed in the vehicle/sham and PTE/sham groups. In the tFI groups, locomotor activity was evidently increased in the vehicle/tFI group. In the PTE/tFI groups, locomotor activity shown in the 50 mg/kg and 100 mg/kg PTE/tFI groups was similar to that in the vehicle/tFI group, but locomotor activity observed in the 200-mg/kg PTE/tFI group was not significantly increased when compared with that shown in the 50 mg/kg and 100 mg/kg PTE/sham groups.

### 3.3. CV Stainability after tFI Was Conserved by PTE 200 mg/kg

**Figure 4:**
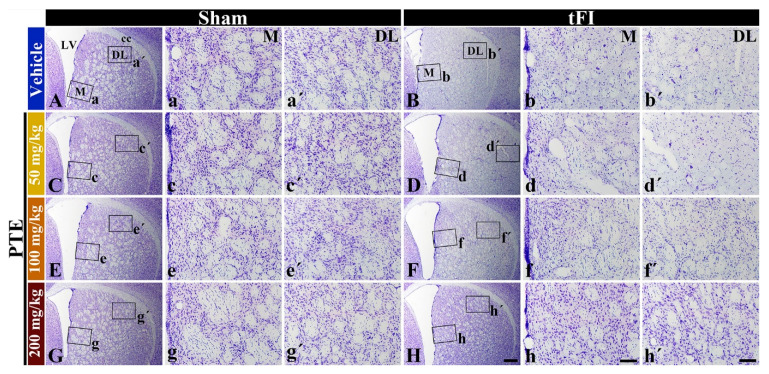
CV staining in the striatum of the vehicle/sham (**A**,**a**,**a’**), vehicle/tFI (**B**,**b**,**b**’), PTE (50, 100, and 200 mg/kg)/sham (**C**,**c**,**c’**,**E**,**e**,**e’**,**G**,**g**,**g’**), and PTE (50, 100, and 200 mg/kg)/tFI (**D**,**d**,**d’**,**F**,**f**,**f’**,**H**,**h**,**h’**) groups at five days after sham and tFI. In the vehicle/tFI group, CV stainability is remarkably reduced in the medial (M) and dorsolateral (DL) fields. In the 50- and 100-mg/kg PTE/tFI groups, CV stainability is similar to that shown in the vehicle/sham group. However, in the 200-mg/kg PTE/TFI group, CV stainability is significantly conserved. LV, lateral ventricle. Scale bars = 400 μm (**A**–**H**) and 100 μm (**a**–**h**, **a’**–**h’**).

In all sham groups, cells stained with CV were easily distinguished throughout the striatum (Figure 4A,C,E,G,Aa,Aa’,Cc,Cc’,Ee,Ee’,Gg,Gg’). In the vehicle/tFI group, a significant loss in CV positive (CV^+^) cells was found in the medial and dorsolateral parts at five days after tFI (Figure 4B,Bb,Bb’). In the 50- and 100-mg/kg PTE/tFI groups, CV stainability was not significantly different from that shown in the vehicle/tFI group (Figure 4D,Dd,Dd’,Ff,Ff’,Hh,Hh’). However, in the 200-mg/kg PTE/tFI group, CV stainability with cells in the medial and dorsolateral parts was similar to that found in the sham groups (Figure 4H,Hh,Hh’). This finding means that striatal cells were well protected from tFI when 200-mg/kg PTE was treated.

### 3.4. F-JB^+^ (Dead) Cells Following tFI Were Significantly Reduced by PTE 200 mg/kg

**Figure 5:**
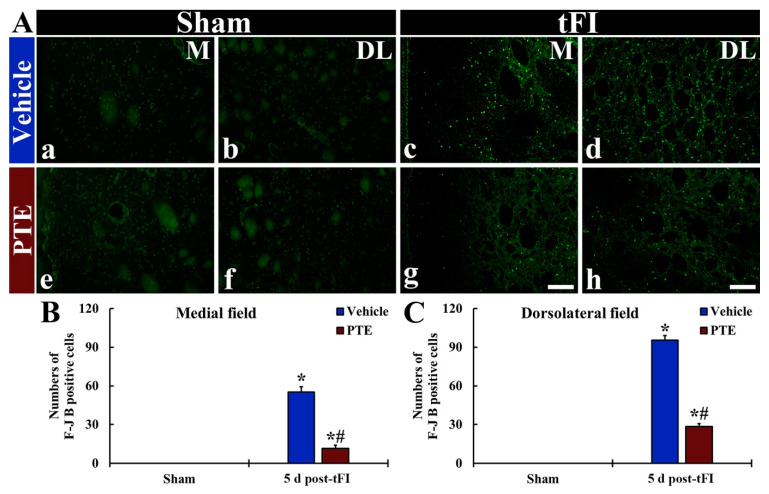
(**A**) FJB histofluorescence in the medial part (**a**–**d**) and dorsolateral part (**e**–**h**) of the striatum of the vehicle/sham (**a**,**b**), vehicle/tFI (**c**,**d**), 200-mg/kg PTE/sham (**e**,**f**), and 200-mg/kg PTE/tFI (**g**,**h**) groups at five days after sham or tFI. In the vehicle/tFI group, F-JB^+^ cells are remarkably increased in the medial (M) and dorsolateral (DL) parts. However, in the 200-mg/kg PTE/tFI group, F-JB^+^ cells are significantly reduced compared with those in the vehicle/tFI group. Scale bar = 50 µm. (**B** and **C**) Numbers of F-JB^+^ cells in the medial (**B**) and dorsolateral (**C**) parts of the striatum. The bars indicate the means ± SEM (*n* = 7; * p < 0.05, significantly different from vehicle/sham group, *^#^ p* < 0.05, significantly different from vehicle/tFI group).

In all sham groups, F-JB^+^ cells (dead cells) were not found throughout the striatum (Figure 5Aa,Ab,Ae,Af). In the vehicle/tFI group, numerous F-JB^+^ cells were shown in the medial and dorsolateral parts of the striatum at five days after tFI (Figure 5Ac,Ad). However, in the 200-mg/kg PTE/tFI group, the numbers of F-JB^+^ cells were significantly reduced in the medial and lateral parts compared with those shown in the vehicle/tFI group (21.2% and 29.9% in the medial and dorsolateral part, respectively, versus the vehicle/tFI group) (Figure 5Ag,Ah,B,C).

### 3.5. PTE 200 mg/kg Protected tFI-induced Damage of Astrocytes

**Figure 6:**
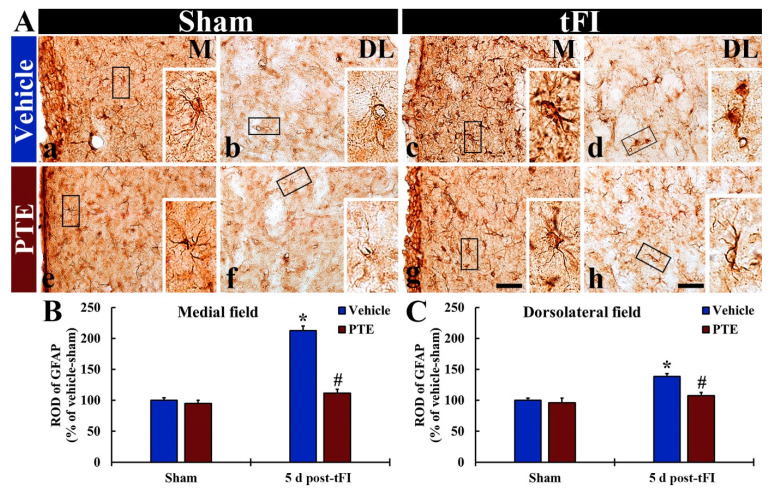
(**A**) IHC for GFAP in the striatum of the vehicle/sham (**a**,**b**), vehicle/tFI (**c**,**d**), 200-mg/kg PTE/sham (**e**,**f**), and 200-mg/kg PTE/tFI (**g**,**h**) groups at five days after sham or tFI. In the vehicle/tFI group, GFAP^+^ astrocytes are damaged (swollen cell body and destroyed processes) in the medial (M) and dorsolateral (DL) parts. However, in the 200 mg/kg PTE/tFI group, GFAP^+^ astrocytes are similar to those found in the vehicle/sham group. (**B**,**C**) ROD of GFAP^+^ astrocytes in the medial (**B**) and dorsolateral (**C**) parts of the striatum. The bars indicate the means ± SEM (*n* = 7; * p < 0.05, significantly different from vehicle/sham group, *^#^ p* < 0.05, significantly different from vehicle/tFI group).

Intact GFAP^+^ astrocytes in the vehicle/sham group were found in the medial and dorsolateral parts of the striatum, and they had small cell body and thread-like (thin) processes (Figure 6Aa,Ab). In the vehicle/tFI group, the cell bodies of GFAP^+^ astrocytes were swollen, and their processes were destroyed (short and thickened) at five days after tFI. In this group, the ROD of GFAP^+^ structures was significantly increased compared with that shown in the vehicle/sham group (212% and 138% in the medial and dorsolateral part, respectively, versus the vehicle/sham group) (Figure 6Ac,Ad,B,C). In the 200-mg/kg PTE/sham group, the morphology and ROD of GFAP^+^ astrocytes were not different from those found in the vehicle/sham group (Figure 6Ae,Af,B,C). Whereas, in the 200-mg/kg PTE/tFI group, GFAP^+^ astrocytes were significantly less damaged in the medial and dorsolateral parts (Figure 6Ag,Ah). In this group, the ROD was significantly low when compared with that shown in the vehicle/tFI group (52.3% and 77.8% in the medial and dorsolateral part, respectively, versus the vehicle/tFI group) (Figure 6B,C).

### 3.6. PTE 200 mg/kg Preserved AEF from tFI Injury

**Figure 7:**
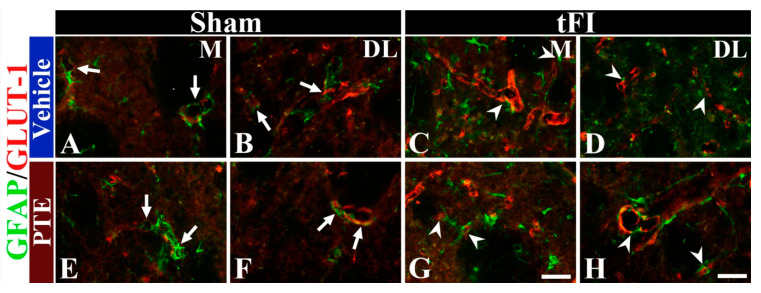
Double immunohistofluorescence for GFAP (green) and GLUT-1 (red) in the striatum of the vehicle/sham (**A**,**B**), vehicle/tFI (**C**,**D**), 200-mg/kg PTE/sham (**E**,**F**), and 200-mg/kg PTE/tFI (**G**,**H**) groups at five days after sham or tFI. In the vehicle/sham group, GFPA^+^ AEF (arrows) are easily shown in the medial (M) and dorsolateral (DL) parts. In the vehicle/tFI group, GFPA^+^ AEF are damaged (arrowheads); however, in the 200-mg/kg PTE/tFI group, GFAP^+^ AEF are preserved (white arrowheads). Scale bar = 50 μm.

AEF, which is a component of BBB, was examined in all groups by double immunofluorescence for GFAP (a marker for astrocyte) and GLUT-1 (a marker for endothelial cells) antibodies (Figure 7). In all sham groups, GFAP^+^ AEF wrapped around GLUT-1^+^ endothelial cells (Figure 7A,B,E,F). In the vehicle/tFI group, GFAP^+^ AEF did not wrap around GLUT-1^+^ structures well at five days after tFI (Figure 7C,D); this indicates that BBB structure was disrupted after tFI. However, in the 200-mg/kg PTE/tFI group, the distribution pattern of GFAP^+^ AEF and GLUT-1^+^ endothelial cells was similar to that found in the sham groups (Figure 7G,H); this indicates that AEF were preserved from tFI injury.

### 3.7. IgG Leakage Following tFI Was Protected by PTE 200 mg/kg

**Figure 8:**
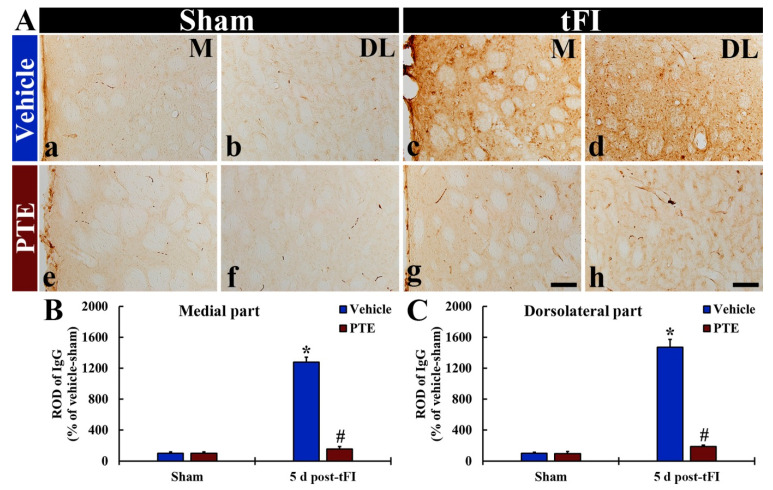
(**A**) IHC for IgG in the striatum of the vehicle/sham (**a**,**b**), vehicle/tFI (**c**,**d**), 200-mg/kg PTE/sham (**e**,**f**), and 200 mg/kg-PTE/tFI (**g**,**h**) group at five days after sham or tFI. In the vehicle/ischemia group, IgG immunoreactivity remarkably increased in the medial (M) and dorsolateral (DL) parts. However, in the 200-mg/kg PTE/tFI group, IgG immunoreactivity is significantly low compared with that in the vehicle/tFI group. Scale bar = 50 µm. (**B**,**C**) ROD of IgG immunoreactivities in the medial (**B**) and dorsolateral (**C**) parts of the striatum. The bars indicate the means ± SEM (*n* = 7; * p < 0.05, significantly different from vehicle/sham group, *^#^ p* < 0.05, significantly different from vehicle/tFI group).

In all sham groups, IgG immunoreactivity was hardly found throughout the parenchyma of the striatum, but IgG immunoreactivity was easily detected inside blood vessels; this indicates that BBB was intact (Figure 8Aa,Ab,Ae,Af). In the vehicle/tFI group, IgG immunoreactivity was significantly enhanced (1281.0% and 1472.8% in the medial and dorsolateral part, respectively, versus the vehicle/sham group) at five days after tFI, compared to that shown in the vehicle/sham group (Figure 8Ac,Ad,B,C). In the 200-mg/kg PTE/tFI group, however, IgG immunoreactivity in the striatum dramatically decreased (12.2% and 12.8% in the medial and dorsolateral part, respectively, versus the vehicle/tFI group) compared with that shown in the vehicle/tFI group (Figure 8Ag,Ah,B,C).

## 4. Discussion

The genus *Populus* (poplar) is well known to contain phenolic compounds and flavonoids, which are main components of poplar extracts and related to various pharmacological activities [1]. In addition, many studies have reported that *Populus* species display antioxidant, anti-inflammatory, and hepato-protective activities [2,3,42,43]. In particular, polyphenols contained in plants are dietary components that play various pharmacological effects. Great interest has been focused on polyphenols because they exert antioxidant and anti-inflammatory activities [44]. It is well known that oxidative stress must be a main event in the pathogenesis of brain ischemia. The overproduction of reactive oxygen species during ischemia and reperfusion damage nucleic acids, lipids, and proteins, thereby leading to neuronal cell death [42,44].

There are evidences supporting the hypothesis that polyphenols-containing plants provide protection against brain ischemia associated changes [42,44]. Cerebral stroke including ischemic insults is one of the main causes of morbidity and mortality in the world [43,45,46]. Unfortunately, however, no new drugs have come up despite numerous efforts of drug development. In our current study, we found that PTE contained various phenolic compounds (gallic acid, catechin, chlorogenic acid, caffeic acid, *p*-coumaric acid). Thereby, this experiment was carried out to examine the effects of PTE in gerbil striatum following 15-min tFI.

We, in the present study, found that locomotor hyperactivity in ischemic gerbils increased after one day of tFI. This experiment was carried out based on many papers which showed that locomotor activity was affected at 1 day after tFI in gerbils [47,48,49]. Pretreatment with 50 and 100 mg/kg of PTE did not reduce the locomotor hyperactivity by tFI, but pretreatment with 200 mg/kg of PTE significantly decreased the locomotor hyperactivity compared with that shown in the vehicle/sham group. This finding suggested that 200 mg/kg of PTE had neuroprotective effects against tFI-induced injuries. Thereby, we examined neuroprotection in the striatum after tFI and the protective effects of 200 mg/kg of PTE.

The tFI model is commonly used to understand tFI-induced behavioral impairment, neuronal damage/death, and their mechanisms after ischemic insults in the forebrain [50,51,52]. Until now, there are numerous studies on tFI-induced neuronal damage and its mechanism in the hippocampus using rodents; however, studies in the basal ganglia are much less frequent than those in the hippocampus. The striatum (also called neostriatum) is the largest nucleus of the basal ganglia (nucleus) and receives glutamatergic inputs from the cerebral cortex and dopaminergic inputs from the substantia nigra located in the midbrain [16,17]. It has been reported that the dorsolateral striatum is susceptible to tFI in rats [18] and gerbils [19].

First of all, we found that numerous striatal cells were positive to F-JB at five days after 15-min tFI. This finding means that striatal cells in gerbils die at five days post-tFI; this is also strongly supported by previous reports that showing that striatal neurons are damaged or dead by at least 15-min tFI [14,15]. In addition, we examined the neuroprotective effect of PTE. When the lower doses of PTE (50 or 100 mg/kg) were administered to gerbils before tFI induction, no neuroprotective effect was found. However, when 200 mg/kg of PTE was used, striatal cells were protected from TFI injury. Therefore, pre-treatment with PTE before tFI provided significant neuroprotection against tFI in gerbil striatum after 15-min tFI. Based on this result, we determined that at least 200 mg/kg of PTE should be required to protect striatal neurons from tFI.

Astrocytes are the most common glial cell type in the CNS (brain and spinal cord) and perform many functions, including the support of endothelial cells to constitute BBB, the maintenance of extracellular ion balance, the provision of nutrients to nervous tissues, and participation in the repair of the CNS following injuries [53,54]. Astrocytes carry out many important roles in both healthy and injured CNS; thus, they have become a potential treatment target for ischemic stroke [55,56,57]. Morphological and functional characteristics of astrocytes are changed in the CNS under pathological conditions, a process termed “astrogliosis”. Astrogliosis is characterized by hypertrophy, proliferation, and increased expression of GFAP [35,58]. An increase in GFAP-positive cells does no result from the generation of new astrocytes, but rather from an increase in GFAP synthesis and a condensation of glial filaments in pre-existing cells [59]. In addition, the reactivity of GFAP under pathological conditions depends on the degree of astrocyte damage [35,60,61,62]. For instance, in transient brain ischemia, a longer ischemic duration, or transient ischemia under higher temperature leads to much severer astrogliosis in damaged sites [63]. In our current study, GFAP^+^ astrocytes in the striatum showed strong GFAP immunoreactivity and were swollen with destroyed (short and thickened) processes at five days after tFI; however, treatment with 200 mg/kg of PTE significantly alleviated astrogliosis in the striatum following tFI. In particular, in our current study, GFAP^+^ AEF did not wrap around GLUT-1^+^ structures (endothelial cells) well at five days after tFI, but treatment with PTE well preserved AEF-enclosing microvessels at five days after tFI. For reference on AEF, the AEF of astrocytes envelop almost all (>99%) of the endothelium [64]. It has been reported that, in a gerbil model of tFI, microvessels enclosed by AEF are significantly reduced in the hippocampal CA1 region after 5-min tFI [65]. Taken together, our findings suggest that the increase in GFAP immunoreactivity in astrocytes in the ischemic striatum is related with an uptake of harmful substances following tFI injury and that this astrogliosis is attenuated or protected by PTE, which may be associated with neuroprotection.

Finally, we found that the extravasation of IgG into the striatal parenchyma was apparently found at five days after tFI, but the extravasated IgG was not shown in the ischemic striatum treated with PTE. The disruption of BBB is known as one of the common pathological features in ischemic stroke [66,67]. Assessment of the leakage of IgG is widely used to determine BBB disruption following brain ischemia [24,25,27,28,68]. In ischemic brain tissue, blood-derived substances can permeate damaged BBB and leads to vasogenic edema and hemorrhagic transformation [69]. It has been reported that IgG extravasation is significantly increased in ipsilateral cerebral cortex following transient focal ischemia induced by occlusion of the middle cerebral artery in rats [70]. In addition, IgG immunoreactivity is significantly increased in the hippocampal CA1 region at five days following tFI [34]. Furthermore, IgG leakage in ischemic gerbil hippocampus occurs early and is severe following 15-min tFI compared with that following 5-min tFI [35]. In our current study, we found that strong IgG immunoreactivity was shown in the striatum at five days after tFI, but the extravasation of IgG was prevented by treatment with PTE. This suggests that PTE can be a candidate to protect BBB from ischemic insults.

In this study, we did not examine the mechanisms of protection of neurons and astrocytes BBB from ischemic injury induced by 15-min tFI. Some studies have demonstrated the antioxidant efficacy of PTE. For some examples, pre-treatment with PTE protected neuronal death/loss in the hippocampal CA1 region against 5-min tFI in gerbils showed that pre-treated PTE markedly increased both SOD1 and SOD2 in the hippocampal CA1 region [31]. In addition, the rats treated with PTE contained pellets for four weeks lead to significant increases of antioxidant enzymes, such as Cu, Zn-superoxide dismutase (SOD1), Mn-superoxide dismutase (SOD2), catalase, and glutathione peroxidase in their livers and kidneys as compared with those shown in the rats treated with normal diet pellets. In particular, elevations are higher in the rats treated with pellets containing ascorbic acid [68]. Based on these findings, we will examine the mechanisms of protection of neurons and astrocytes BBB from ischemic injury induced by tFI.

## 5. Conclusions

In this study, we found that phenolic compounds, which are known to strongly exert antioxidant activity, were rich in PTE. With this, we examined the effects of PTE on neuronal damage, astrogliosis, and leakage from BBB in gerbil striatum following 15-min tFI. First of all, 200 mg/kg of PTE effectively prevented the death of striatal neurons, which were dead at five days after tFI. For astrogliosis, the tFI caused severe astrocyte damage including the complete loss of AEF wrapping blood vessels, as a component of BBB, but the tFI-induced astrocyte damage was effectively protected by PTE treatment. Simultaneously, strong IgG leakage in the striatal parenchyma at five days after the tFI was hardly observed in the gerbils treated with PTE. Taken together, our current findings suggest that the root of *Populus tomentiglandulosa* has various phenolic compounds and can be employed as an excellent candidate to develop a preventive material against ischemic injury in brains.

## Figures and Tables

**Table 1:** Phenolic compositions of PTE.

	PTE (mg/g)
Gallic acid	1.4 ± 0.35
Catechin	9.1 ± 0.27
Chlorogenic acid	1.6 ± 0.86
Caffeic acid	4.1 ± 0.57
*p*-coumaric acid	2.1 ± 0.47
Ferulic acid	–

## Data Availability

The data presented in this study are available on request from the corresponding author.

## Sample Availability

Samples of the compounds are not available from the authors.

## Abbreviations

AEF: astrocyte endfeet; BCCAO, bilateral occlusion of the common carotid artery; BBB, blood–brain barrier; CNS, central nervous system; CA, cornu ammonis; CV, cresyl violet; DND, delayed neuronal death; F–J B, Fluoro–Jade B; GFAP, glial fibrillary acidic protein; GLUT–1, glucose transporter 1; HPLC, high–performance liquid chromatography; IgG, immunoglobulin G; PTE, *Populus tomentiglandulosa* extract; SMA, Spontaneous motor activity; tFI, transient forebrain ischemia
